# TECHNOLOGY-ASSISTED TOILETING BY OLDER ADULTS: A SCOPING REVIEW OF BIDET USE

**DOI:** 10.1093/geroni/igae098.2566

**Published:** 2024-12-31

**Authors:** Catherine Van Son, Minyoung Cerruti

**Affiliations:** Washington State University, MARYSVILLE, Washington, United States; Washington State University, Pullman, Washington, United States

## Abstract

**Objective:**

With aging, toileting can become difficult, given changes in mobility, agility, and cognition. Interventions are needed to support toileting privacy and independence. A technology-assistive device for toileting, referred to as a bidet (washlet), uses one’s existing toilet and plumbing to provide a cleansing mechanism by spraying water on the perineum after toileting. Using a remote-controlled device, water temperature, and pressure can be individualized, and the seat warmed. This review focused on the following question: What are the benefits and challenges older adults (OA) and their caregivers experience in using a bidet?

**Methods:**

A scoping review was conducted using the five stages of Arksey and O’Malley’s framework and the PRISMA-ScR checklist. Our search focused on literature published after 2000 and included five databases.

**Results:**

Fourteen articles were identified and analyzed. Seven papers focused on OAs, two on caregivers for OAs, and five included OAs as part of their sample. Findings described bidet technology facilitators and barriers for OAs and their caregivers. Older adults described apprehension about using the technology while appreciating increased privacy and independence. Caregivers reported less time toileting and could focus more on other ADL activities. Research gaps were identified, and recommendations were proposed to change the culture of toileting and promote bidet use. Randomized controlled studies with larger samples are needed to determine the feasibility and effectiveness of bidet use by older adults.

**Conclusion:**

Bidet technology can support OAs’ toileting privacy and independence, reduce caregiver burden, and decrease the environmental impact by using fewer paper products.

